# Plasma copeptin, kidney disease, and risk for cardiovascular morbidity and mortality in two cohorts of type 2 diabetes

**DOI:** 10.1186/s12933-018-0753-5

**Published:** 2018-08-02

**Authors:** Gilberto Velho, Stéphanie Ragot, Ray El Boustany, Pierre-Jean Saulnier, Mathilde Fraty, Kamel Mohammedi, Frédéric Fumeron, Louis Potier, Michel Marre, Samy Hadjadj, Ronan Roussel

**Affiliations:** 1 INSERM, UMRS 1138, Centre de Recherche des Cordeliers, 15 rue de l’École de Médecine, Paris, 75006 France; 2 INSERM, CIC 0802, Poitiers, France; 3 UFR de Médecine et Pharmacie, Université de Poitiers, Poitiers, France; 4 INSERM, Research Unit 1082, Poitiers, France; 5 Service d’Endocrinologie, Diabétologie, Nutrition, Hôpital Haut-Lévêque, Pessac, France; 6 Faculté de Médecine Paul Broca, Université de Bordeaux, Bordeaux, France; 7 Department of Diabetology, Endocrinology and Nutrition, Assistance Publique–Hôpitaux de Paris (AP-HP), Bichat Hospital, DHU FIRE, Paris, France; 8 Université Paris Diderot, Sorbonne Paris Cité, UFR de Médecine, Paris, France; 9 Department of Endocrinology and Diabetology, Centre Hospitalier Universitaire de Poitiers, Poitiers, France

**Keywords:** Copeptin, Vasopressin, Type 2 diabetes, Cardiovascular disease, Diabetic kidney disease, Epidemiology

## Abstract

**Background:**

Cardiovascular disease and kidney damage are tightly associated in people with type 2 diabetes. Experimental evidence supports a causal role for vasopressin (or antidiuretic hormone) in the development of diabetic kidney disease (DKD). Plasma copeptin, the COOH-terminal portion of pre-provasopressin and a surrogate marker of vasopressin, was shown to be positively associated with the development and progression of DKD. Here we assessed the association of plasma copeptin with the risk of cardiovascular events during follow-up in two prospective cohorts of type 2 diabetic patients, and we examined if this association could be mediated by deleterious effects of vasopressin on the kidney.

**Methods:**

We studied 3098 and 1407 type 2 diabetic patients from the French cohorts DIABHYCAR and SURDIAGENE, respectively. We considered the incidence during follow-up (median: 5 years) of a combined end point composed of myocardial infarction, coronary revascularization, hospitalization for congestive heart failure, or cardiovascular death. Copeptin concentration was measured in baseline plasma samples by an immunoluminometric assay.

**Results:**

The cumulative incidence of cardiovascular events during follow-up by sex-specific tertiles of baseline plasma copeptin was 15.6% (T1), 18.7% (T2) and 21.7% (T3) in DIABHYCAR (p = 0.002), and 27.7% (T1), 34.1% (T2) and 47.6% (T3) in SURDIAGENE (p < 0.0001). Cox proportional hazards survival regression analyses confirmed the association of copeptin with cardiovascular events in both cohorts: hazard ratio with 95% confidence interval for T3 vs. T1 was 1.29 (1.04–1.59), p = 0.02 (DIABHYCAR), and 1.58 (1.23–2.04), p = 0.0004 (SURDIAGENE), adjusted for sex, age, BMI, duration of diabetes, systolic blood pressure, arterial hypertension, HbA1c, total cholesterol, HDL-cholesterol, triglycerides, estimated glomerular filtration rate (eGFR), urinary albumin concentration (UAC), active tobacco smoking, and previous history of myocardial infarction at baseline. No interaction was observed between plasma copeptin and eGFR (p = 0.40) or UAC (p = 0.61) categories on the risk of cardiovascular events in analyses of pooled cohorts.

**Conclusions:**

Plasma copeptin was positively associated with major cardiovascular events in people with type 2 diabetes. This association cannot be solely accounted for by the association of copeptin with kidney-related traits.

**Electronic supplementary material:**

The online version of this article (10.1186/s12933-018-0753-5) contains supplementary material, which is available to authorized users.

## Background

Cardiovascular disease (CVD) is a major cause of morbidity and mortality in people with type 2 diabetes [1], who have a threefold higher risk than nondiabetic individuals of developing atherosclerosis and its clinical complications [2, 3]. Moreover, about 20–30% of patients with type 2 diabetes develop diabetic kidney disease (DKD) [4], which is also associated with increased risk of cardiovascular morbidity and mortality [5].

An increasing body of experimental, pharmacological and epidemiological data supports a causal role of vasopressin (or antidiuretic hormone) in the development and progression of chronic kidney disease (CKD) [6–17]. Plasma copeptin, the COOH-terminal portion of pre-provasopressin and a surrogate marker of vasopressin, was shown to be positively associated with the decline in kidney function in the general population [11, 12, 17], and with the development and progression of DKD in type 1 and type 2 diabetes [10, 13, 16]. Plasma copeptin was also shown to be associated with CVD in a few studies [18–20], especially in elder people with diabetes [19, 20].

Here, we investigated the association of baseline plasma copeptin with the incidence of CVD in two independent French cohorts of people with type 2 diabetes. We looked for interactions between plasma copeptin and markers of kidney function at baseline in the association of copeptin with CVD risk to evaluate if this association could be accounted for by deleterious effects of vasopressin on the kidney.

## Methods

### Study population

We studied 3098 and 1407 type 2 diabetic subjects from the DIABHYCAR and SURDIAGENE cohorts, respectively. DIABHYCAR was a multinational, multicentric clinical trial conducted in people with type 2 diabetes selected on the basis of persistent microalbuminuria (urinary albumin concentration, UAC = 20–200 mg/l) or macroalbuminuria (UAC > 200 mg/l) without renal failure (plasma creatinine < 150 µmol/l) at baseline [21]. Patients in the French branch of DIABHYCAR (included in the present investigation) were followed and recruited into the study by general practitioners. The trial tested the effect of a low dose of ramipril, an angiotensin converting enzyme (ACE) inhibitor, on the incidence of cardiovascular and/or renal events. The median duration of follow-up was 4.7 years. Results were negative regarding the drug effect and were published previously [22]. SURDIAGENE is an ongoing prospective monocentric study aiming to identify the genetic and environmental determinants of vascular complications in type 2 diabetes [23, 24]. Patients have been recruited and followed regularly since 2002 at the diabetes department of the University Hospital of Poitiers, France. Living status and cardiovascular and kidney end points were determined from patients’ hospital records and interviews with general practitioners, and recorded every other year since 2007. Median duration of follow-up was 5 years. Detailed description of study population, outcome criteria, and adjudication procedure was published previously for both cohorts [21, 22, 25].

### Definition of clinical parameters and outcomes

Arterial hypertension was defined as systolic blood pressure (SBP) > 140 mmHg and/or diastolic blood pressure (DBP) > 90 mmHg, or SBP and DBP below these values in the presence of antihypertensive medication and history of hypertension. We considered the incidence of cardiovascular events during follow-up, a combined end point composed of coronary heart disease (myocardial infarction or coronary revascularization), congestive heart failure (CHF) or cardiovascular death. Myocardial infarction was diagnosed as the occurrence of at least 2 out of 3 of the following criteria: constrictive chest pain lasting 20 min or longer, increased serum creatine phosphokinase activity and/or troponin concentration, or typical electrocardiographic changes. Coronary revascularization included cases of coronary artery bypass grafting and percutaneous coronary intervention. Incident cases of CHF were defined as the occurrence during follow-up of hospitalization due to CHF as defined by the 2012 criteria of the European Society of Cardiology [26]. Cardiovascular death was defined as death following myocardial infarction, CHF, arrhythmias or stroke. In both cohorts the outcomes were prospectively collected (except for CHF data, readjudicated in 2014), and were adjudicated by independent ad hoc committees [21, 22, 25].

Estimated glomerular filtration rate (eGFR) was calculated using the CKD-EPI study equation [27]. eGFR categories were defined as proposed by the Kidney Disease Improving Global Outcomes (KDIGO) group [28]: GFR ≥ 90 ml/min/1.73 m^2^ (G1), 90 > GFR ≥ 60 ml/min/1.73 m^2^ (G2), 60 > GFR ≥ 45 ml/min/1.73 m^2^ (G3A), 45 > GFR ≥ 30 ml/min/1.73 m^2^ (G3B), 30 > GFR ≥ 15 ml/min/1.73 m^2^ (G4), GFR < 15 ml/min/1.73 m^2^ (G5). The slope of eGFR variation during follow-up was computed for each individual using the simple linear regression coefficient determined from all baseline and follow-up values. Rapid kidney function decline during follow-up was defined as a slope of eGFR steeper than − 5 ml/min/1.73 m^2^ per year [28]. We also considered the incidence of a kidney outcome defined as doubling of serum creatinine or the development of ESRD (requirement of hemodialysis or kidney transplantation) during follow-up.

### Laboratory procedures

Copeptin concentration was measured in fasting plasma-EDTA samples, collected at baseline and kept frozen at − 80 °C. Copeptin measurements were performed by ThermoFisher Scientific using their automated immunoluminometric assay (ultra-sensitive B•R•A•H•M•S Copeptin proAVP, Thermo Scientific, Hennigsdorf, Germany) [29, 30]. The limit of detection was 0.9 pmol/l. Intra-assay CV reported by the manufacturer was < 15 and < 8% for concentrations range of 2.0–4.0 and 4.0–15.0 pmol/l, respectively. Inter-assay CV was < 18% and < 10%, respectively, for the lower and higher copeptin concentration range. Urinary albumin was measured by nephelometry. Biobanking and laboratory procedures for blood biochemistry assays in both cohorts are described elsewhere [22, 25].

### Computations and statistical analyses

Cohort and sex-specific tertiles of plasma copeptin concentration were computed to take into account cohort-related and the well-known sex-related differences in copeptin levels [31–35]. Results are expressed as mean ± SD, except where stated otherwise. Differences between groups were assessed by Pearson’s Chi squared test, Wilcoxon/Kruskal–Wallis test, ANOVA or ANCOVA. Kaplan–Meier curves were used to plot the incidence of outcomes over time. Cox proportional hazards survival regression analyses and logistic regression analyses were used to examine the effect of plasma copeptin at baseline on outcomes during follow-up and to evaluate the independence of this association from other relevant covariates, and specially from markers of kidney function. Hazard ratios (HR) or Odds ratios (OR) with their 95% confidence intervals (CI) were computed in these analyses. The association of plasma copeptin with the risk of cardiovascular events was tested in two sets of analyses. In the first set, data from the cohorts were analyzed separately and two regression models were tested in each cohort. Model 1 included as independent covariates baseline parameters with p < 0.10 (in at least one of the cohorts) in the comparison between incident cases and participants with no events (data from Table 1), except UAC and eGFR, while model 2 also included these markers of kidney function. The second set of analyses was performed with pooled data from the cohorts to increase sample size and the number of events during follow-up, and thus the statistical power of the analyses. Cohort membership was always included as a covariate in the regression models to take into account cohort-related differences. We looked for associations of baseline plasma copeptin with the incidence of individual cardiovascular outcomes (myocardial infarction, coronary revascularization, CHF, cardiovascular death, stroke) during follow-up. We also tested interactions between the copeptin tertiles and KDIGO eGFR categories or UAC categories (normo, micro or macroalbuminuria) in the association of copeptin with cardiovascular events. For standardization of results, quantitative covariates were expressed as qualitative dichotomous (below or above the cohort-specific median) variables, and the interactions were assessed by including in the regression models interaction terms (“copeptin tertile/KDIGO eGFR category” and “copeptin tertile/UAC category”). For all analyses, data were log-transformed when the normality of the distribution was rejected by the KSL goodness of fit test. Statistics were performed with JMP (SAS Institute Inc., Cary, NC). P < 0.05 was considered as significant.

**Table 1:** Clinical characteristics at baseline by the incidence of cardiovascular events during follow-up

	DIABHYCAR		p	SURDIAGENE		p
	No events	Incident cases		No events	Incident cases	
N	2521	577		894	513	
Sex: male (%)	72	79	0.0003	55	63	0.006
Age (years)	65 ± 8	68 ± 8	< 0.0001	63 ± 11	69 ± 10	< 0.0001
BMI (kg/m^2^)	29.5 ± 4.6	28.8 ± 4.5	0.0006	31.7 ± 6.5	30.8 ± 5.8	0.01
Duration of diabetes (years)	10 ± 8	11 ± 8	0.0003	13 ± 10	17 ± 10	< 0.0001
HbA1c (%)	7.8 ± 1.7	8.1 ± 1.9	0.006	7.7 ± 1.6	7.9 ± 1.5	0.03
HbA1c (mmol/mol)	62 ± 19	65 ± 20	0.007	61 ± 17	63 ± 16	0.02
Systolic blood pressure (mmHg)	145 ± 14	147 ± 14	0.002	131 ± 17	135 ± 19	< 0.0001
Diastolic blood pressure (mmHg)	82 ± 8	82 ± 8	0.87	73 ± 11	72 ± 12	0.40
Arterial Hypertension (%)	55	63	0.0003	79	91	< 0.0001
UAC (mg/l)*	72 (126)	107 (254)	<0.0001	19 (60)	48 (257)	< 0.0001
UAC						
Normoalbuminuria (%)	0	0		51.8	35.7	
Microalbuminuria (%)	78.6	67.4	< 0.0001	36.7	36.4	< 0.0001
Macroalbuminuria (%)	21.4	32.6		11.5	27.9	
Creatinine (µmol/l)	88 ± 20	95 ± 20	< 0.0001	90 ± 59	122 ± 95	< 0.0001
eGFR (ml/min/1.73 m^2^)	78 ± 20	73 ± 19	< 0.0001	78 ± 22	62 ± 27	< 0.0001
KDIGO						
G1 (%)	26.0	17.8	< 0.0001	36.7	17.0	< 0.0001
G2 (%)	56.2	55.5		43.6	38.4	
G3A (%)	15.8	21.8		10.7	19.5	
G3B (%)	2.0	4.9		6.2	10.5	
G4 (%)	0	0		1.6	10.5	
G5 (%)	0	0		1.2	4.1	
Total cholesterol (mmol/l)	5.76 ± 1.06	5.95 ± 1.09	<0.0001	4.75 ± 1.13	4.83 ± 1.22	0.30
HDL cholesterol (mmol/l)	1.33 ± 0.36	1.26 ± 0.32	< 0.0001	1.20 ± 0.41	1.18 ± 0.41	0.34
Triglycerides (mmol/l)	2.19 ± 1.44	2.32 ± 1.29	0.003	1.90 ± 1.30	1.97 ± 1.69	0.81
Previous myocardial infarction (%)	4	12	< 0.0001	9	27	<0.0001
Active tobacco smoking (%)	14	15	0.55	12.0	8.1	0.02
Copeptin (pmol/l)*	7.1 (6.6)	7.9 (7.4)	0.0001	6.2 (7.0)	8.4 (11.4)	< 0.0001

Data expressed as mean ± SD except (*) expressed as median and interquartile range. Statistics for quantitative parameters are ANOVA with log-transformed data, except (*) Wilcoxon/Kruskal–Wallis rank sums test. HbA1c is expressed in % of total hemoglobin and in mmol/mol (millimoles HbA1c per mole of total hemoglobin). KDIGO categories (G1 to G5) are defined by decreasing eGFR values (see “Methods”). UAC: urinary albumin concentration. p < 0.05 is significant

## Results

### Copeptin and cardiovascular events during follow-up

Cardiovascular events comprised 94 cases of myocardial infarction, 290 cases of coronary revascularization, 127 cases of CHF, and 208 cases of cardiovascular death in 577 (18.6%) DIABHYCAR participants. It comprised 121 cases of myocardial infarction, 161 cases of coronary revascularization, 241 cases of CHF, and 297 cases of cardiovascular death in 513 (34.5%) SURDIAGENE participants. The incidence rates were 4.5 and 5.0 per 100 person-years in DIABHYCAR and SURDIAGENE, respectively. Characteristics of participants at baseline by the incidence of cardiovascular events during follow-up are shown in Table 1. Briefly, incident cases of cardiovascular events in each of the cohorts, as compared to participants not presenting the outcome, were more likely to be men, were older, had a longer duration of diabetes, higher systolic blood pressure and lower BMI. They had higher circulating levels of copeptin, HbA1c and UAC, and lower eGFR. Arterial hypertension and a previous history of myocardial infarction were more frequent in incident cases from both cohorts. Triglycerides and total cholesterol were higher, and HDL-cholesterol was lower in incident cases from DIABHYCAR.

**Fig. 1:**
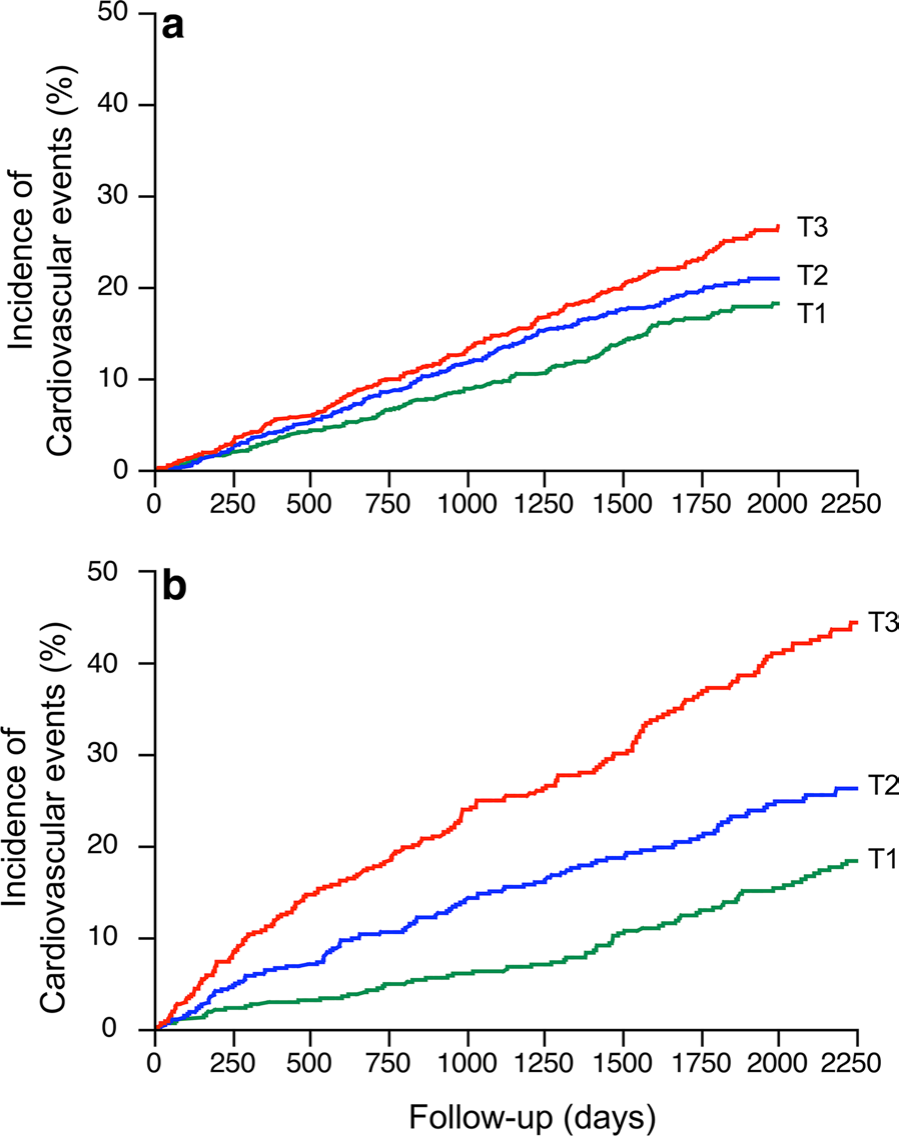
Kaplan-Meier curves for the cumulative incidence of cardiovascular events during follow-up by tertiles of baseline plasma copeptin. **a** DIABHYCAR study; log-rank test Chi square = 15.9, p = 0.0004. **b** SURDIAGENE study; log-rank test Chi square = 78.4, p < 0.0001

Characteristics of participants at baseline by tertiles of plasma copeptin are shown in Additional file 1: Table S1. The cumulative incidence of cardiovascular events during follow-up by tertiles of baseline plasma copeptin was 15.6% (T1), 18.7% (T2) and 21.7% (T3) in DIABHYCAR participants (Pearson’s Chi square 12.6, p = 0.002), and 27.7% (T1), 34.1% (T2) and 47.6% (T3) in SURDIAGENE participants (Pearson’s Chi square 41.3, p < 0.0001). Kaplan–Meier (cumulative incidence) curves for the outcome during follow-up by tertiles of plasma copeptin at baseline are shown in Fig. 1. In DIABHYCAR, Cox analysis with Model 1 (no markers of kidney function as covariates) confirmed a positive association of the upper tertiles of plasma copeptin, and of log_e_[copeptin], with the incidence of cardiovascular events during follow-up (Table 2). When baseline UAC and eGFR were included in the Cox analysis (Model 2), the upper tertile of plasma copeptin remained significant associated with the outcome, and a trend towards association was observed for log_e_[copeptin] (Table 2). In SURDIAGENE, Cox analysis with Models 1 and 2 confirmed the positive association of the upper tertiles of plasma copeptin, and of log_e_[copeptin], with the incidence of cardiovascular events during follow-up (Table 2).

**Table 2:** Cardiovascular events during follow-up by tertiles of plasma copeptin at baseline

	DIABHYCAR		SURDIAGENE	
	No events	Cardiovascular events	No events	Cardiovascular events
T1	873 (84.4%)	161 (15.6%)	339 (72.3%)	130 (27.7%)
T2	841 (81.3%)	193 (18.7%)	309 (65.9%)	160 (34.1%)
T3	808 (78.4%)	223 (21.6%)	246 (52.4)	223 (47.6%)

	HR (95% CI)	p	HR (95% CI)	p
Model 1				
T3 vs T1	1.40 (1.13–1.72)	0.001	2.10 (1.68–2.64)	< 0.0001
T2 vs T1	1.28 (1.04–1.59)	0.02	1.46 (1.15–1.85)	0.002
T3 vs T2	1.09 (0.90–1.33)	0.39	1.44 (1.18–1.78)	0.0005
Log_e_[copeptin]	1.21 (1.06–1.37)	0.004	1.52 (1.37–1.67)	< 0.0001
Model 2				
T3 vs T1	1.29 (1.04–1.59)	0.02	1.58 (1.23–2.04)	0.0004
T2 vs T1	1.25 (1.01–1.54)	0.04	1.39 (1.10–1.77)	0.006
T3 vs T2	1.03 (0.85–1.26)	0.75	1.14 (0.91–1.43)	0.27
Log_e_[copeptin]	1.13 (0.99–1.29)	0.06	1.28 (1.12–1.46)	0.0003

Data expressed as number of cases and (%) by line. Hazards ratio (HR) computed by Cox proportional hazards survival regression analysis for tertiles of plasma copeptin, and for 1 unit of log_e_[copeptin]. Model 1: adjusted for sex, age, BMI, duration of diabetes, systolic blood pressure, arterial hypertension, HbA1c, total cholesterol, HDL-cholesterol, triglycerides, active tobacco smoking, and previous history of myocardial infarction at baseline. In DIABHYCAR, analysis was further adjusted for study treatment (randomization group in the original DIABHYCAR study: ramipril vs placebo) during follow-up. Model 2: model 1 plus adjustments for eGFR and UAC at baseline. p < 0.05 is significant

### Sensitivity analyses: copeptin and risk of individual cardiovascular outcomes during follow-up

The higher tertile of plasma copeptin was significantly associated with greater incidence of myocardial infarction, CHF and cardiovascular death, and with the requirement of coronary revascularization during follow-up when the outcomes were analyzed separately (Additional file 1: Table S2). No association was observed with the incidence of stroke. For these analyses, data from both cohorts were pooled to increase the number of events of each individual outcome.

### Copeptin and kidney function at baseline and during follow-up

Plasma copeptin was associated with markers of kidney function at baseline and with evolution of DKD during follow-up in both cohorts. Copeptin levels increased consistently through KDIGO groups (G1–G5) defined by decreasing levels of eGFR, and increased with the severity of albuminuria at baseline (Additional file 1: Figure S1). Increasing tertiles of plasma copeptin at baseline were associated with steeper yearly decline in eGFR. The slope of eGFR decline by tertiles of plasma copeptin was − 1.15 ± 0.17 (T1), − 1.51 ± 0.17 (T2), and − 2.72 ± 0.18 ml/min/1.73 m^2^ per year (T3) for DIABHYCAR (mean ± SEM, ANCOVA p < 0.0001, adjusted for sex, age, eGFR and UAC at baseline and duration of follow-up). It was − 1.09 ± 0.94 (T1), − 2.88 ± 0.96 (T2), and − 4.42 ± 1.06 ml/min/1.73 m^2^ per year (T3) for SURDIAGENE (ANCOVA p = 0.03). Rapid kidney function decline during follow-up was observed in 432 DIABHYCAR and 258 SURDIAGENE participants. Association of baseline plasma copeptin with rapid kidney function decline was confirmed by logistic regression analyses in both cohorts (Additional file 1: Table S3). Association of baseline plasma copeptin with a kidney outcome defined as doubling of serum creatinine or the development of ESRD during follow-up was previously reported for DIABHYCAR [10], and is shown in Additional file 1: Table S4 for SURDIAGENE.

### Interactions of copeptin and markers of kidney function on cardiovascular risk

**Fig. 2:**
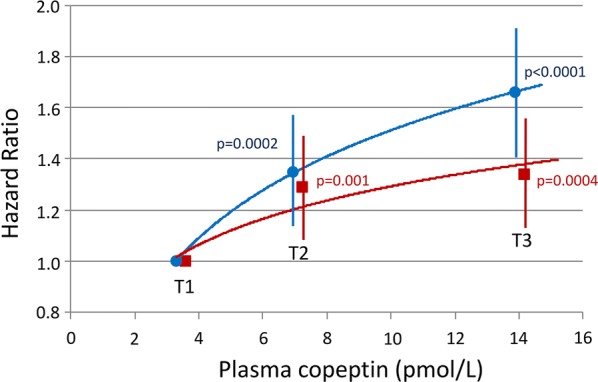
Hazard ratio (HR) with 95% confidence interval, and p-values for tertiles of baseline plasma copeptin (T3 or T2 versus T1), in Cox regression analyses of the incidence of cardiovascular events during follow-up. Pooled data from DIABHYCAR to SURGENE cohorts. Model 1 (blue circles): adjusted for cohort, sex, age, BMI, duration of diabetes, systolic blood pressure, arterial hypertension, HbA1c, total cholesterol, HDL-cholesterol, triglycerides, active tobacco smoking, and previous history of myocardial infarction at baseline. Model 2 (red squares): model 1 plus adjustments for KDIGO eGFR categories and UAC categories (normo, micro or macroalbuminuria) at baseline

**Fig. 3:**
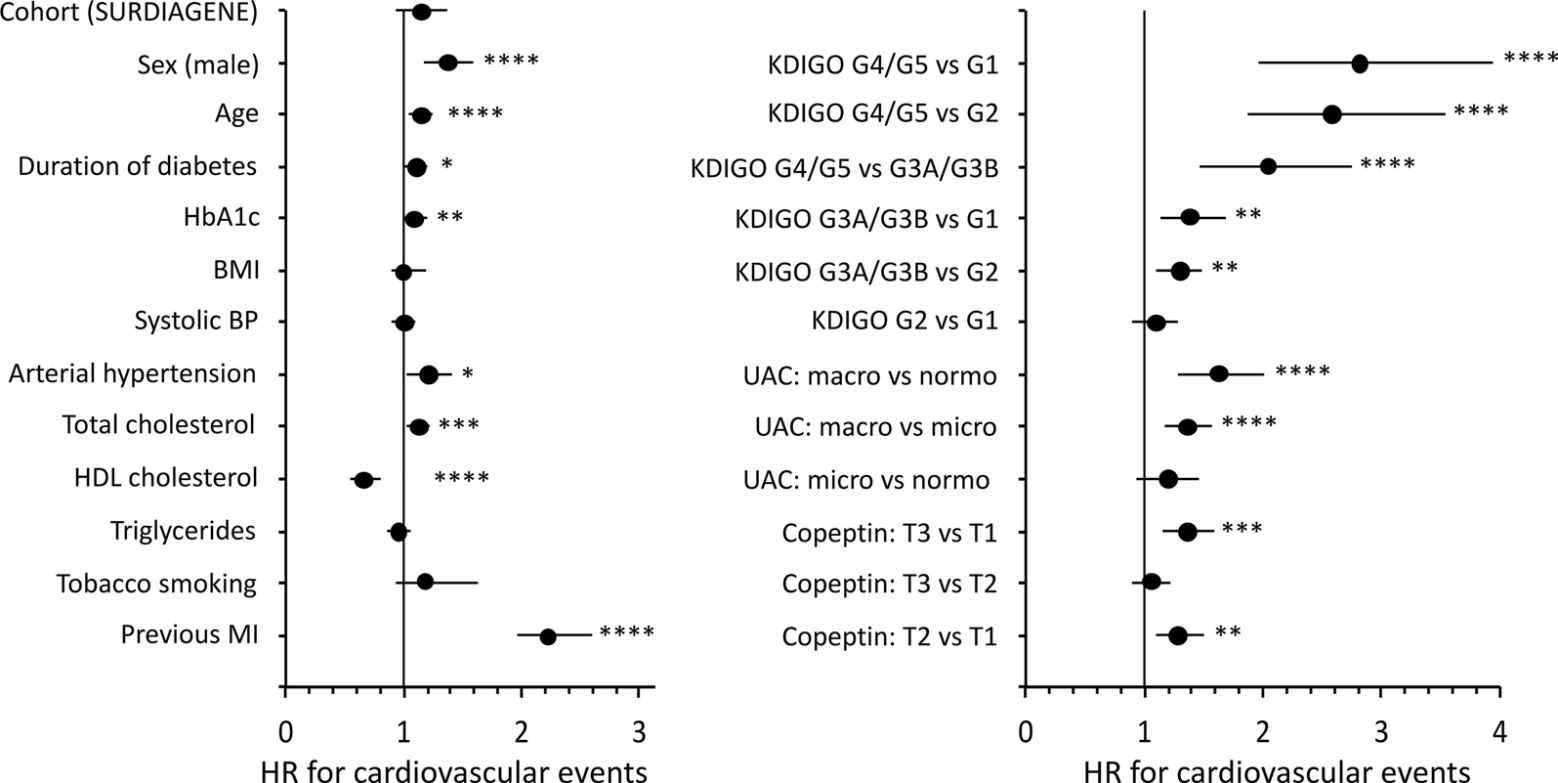
Forest plot showing Hazard Ratio (HR) and 95% confidence interval for baseline covariates included in the Cox regression analysis for the incidence of cardiovascular events during follow-up (Model 2). Pooled data from DIABHYCAR and SURGENE cohorts. Quantitative covariates are expressed as qualitative dichotomous (below or above the median) variables except for UAC (normo-, micro- or macroalbuminuria) and copeptin (expressed as increasing sex and cohort specific tertiles, T1 to T3). MI: myocardial infarction. Estimated glomerular filtration rate (eGFR) calculated with the CKD-EPI equation. KDIGO categories (G1 to G5) defined by decreasing eGFR values (see methods). UAC: urinary albumin concentration. *p < 0.05, **p < 0.01, ***p < 0.001, ****p < 0.0001

Interactions of plasma copeptin and markers of kidney function at baseline on the risk of cardiovascular events during follow-up were tested. Data from the cohorts were pooled for these analyses. The upper tertiles of plasma copeptin, and log_e_[copeptin], were significantly associated with the incidence of cardiovascular events in both model of Cox analyses, excluding or including markers of kidney function (Additional file 1: Table S5 and Fig. 2). Male sex, age, duration of diabetes, HbA1c, systolic blood pressure, arterial hypertension, macro- or microalbuminuria, higher KDIGO categories (decreased eGFR), and a previous history of myocardial infarction also remained positively associated, and BMI and HDL cholesterol inversely associated with the outcome (Fig. 3). We observed no interaction in the associations: p (interaction) = 0.40 for copeptin tertiles/KDIGO eGFR category; p (interaction) = 0.61 for copeptin tertiles/UAC category (normo, micro, or macroalbuminuria).

## Discussion

In the present investigation in two independent cohorts of people with type 2 diabetes, baseline plasma copeptin was positively associated with the incidence of myocardial infarction, coronary revascularization, congestive heart failure, and cardiovascular death during a 5-year follow-up. The stronger association was observed in a hospital-recruited cohort (SURDIAGENE), in which participants had a high cardiovascular risk at baseline (Table 1) and presented a higher incidence of outcomes during follow-up. The association was confirmed in a cohort drawn from the general practice (DIABHYCAR), in which participants, despite having micro- or macroalbuminuria (but not renal failure) at baseline as per inclusion criteria, had a lesser incidence of outcomes during follow-up. Plasma copeptin was associated with markers of kidney function at baseline (eGFR, UAC), and with the decline of kidney function and the progression of CKD during follow-up in both cohorts, as previously reported in people with type 1 or type 2 diabetes [10, 13, 36], as well as in the general population [7, 11, 33]. Kidney function markers were also associated with the incidence of cardiovascular events during follow-up in our study. However, we observed no interaction of baseline copeptin and kidney function markers in the associations with the cardiovascular outcome. The copeptin association with cardiovascular events was also independent from other relevant risk factors such as dyslipidemia, arterial hypertension, the severity and duration of diabetes, and a previous history of myocardial infarction.

To our knowledge, this is the first investigation to evaluate the interaction of kidney function in the association of copeptin with cardiovascular morbidity and mortality in people with type 2 diabetes. In the population-based Malmö Diet and Cancer Study–Cardiovascular cohort, plasma copeptin was associated with a combined end point (similar to the one we used in our study) composed of coronary heart disease, heart failure, and death in people with diabetes but not in non-diabetic individuals [18]. In the British Regional Heart Study, plasma copeptin was associated with increased risk of incident stroke and with cardiovascular mortality in elder men with diabetes, but not in those without diabetes [20]. No independent association was observed with coronary heart disease events in that study [20]. In contrast, copeptin was associated with increased risk of coronary heart disease and cardiovascular mortality both in diabetic and non-diabetic individuals in a study of Swedish elders [19]. However, interaction with markers of kidney function was not assessed in these investigations. In the German Diabetes and Dialysis Study, high plasma copeptin was associated with increased risk for cardiovascular events (myocardial infarction, stroke, cardiovascular death) and for all-cause mortality in type 2 diabetic patients, but all participants had ESRD and were undergoing hemodialysis [37]. It is noteworthy that unlike what was observed in the British [20] and German [37] studies in selected groups of patients with type 2 diabetes, there was no association of copeptin with stroke in our cohorts. No association with stroke was reported in the Swedish studies neither [18, 19]. High plasma copeptin was also associated with cardiovascular and all-cause mortality in Chinese patients with ischemic stroke [38], and with coronary, infectious and all-cause mortality in patients with CKD (but not in subjects with normal renal function) from the German LURIC and 4D studies [39]. Finally, we have previously observed associations of high levels of plasma copeptin with increased risk for ESRD, ischemic heart disease, and cardiovascular and all-cause mortality in two cohorts of people with long-standing type 1 diabetes [13]. In that study, the risks for ischemic heart disease and cardiovascular mortality were influenced by markers of nephropathy, and the association of copeptin with these outcomes was dependent on UAC, eGFR and on arterial hypertension, mostly a consequence of kidney disease in people with type 1 diabetes.

A few recent short term pilot studies showed that increased water intake can significantly decrease plasma copeptin concentration in healthy individuals [40, 41], especially in those with high plasma copeptin at baseline [40], as well as in patients with stage 3 CKD [42]. Coaching to increase water intake compared with coaching to maintain baseline water intake did not significantly slow the decline in kidney function after 1 year in a small randomized intervention study in patients with stage 3 CKD of various etiologies [43]. However, patients in the increased hydration group presented only a modest increase in 24 h urine volume and a small decrease in plasma copeptin during follow-up, and the authors concluded that the study may have been underpowered to detect a clinically important difference [43].

The pathophysiological mechanisms behind the association of copeptin with CVD are probably complex. Vasopressin binds to three different G-protein coupled receptors. V1aR is widely expressed, particularly in vascular smooth muscle cells, hepatocytes, platelets, and the central nervous system. V1bR is expressed in the endocrine pancreas, in cells of the anterior pituitary and throughout the brain. V2R is predominantly expressed in the kidney collecting ducts and in endothelium. A large body of data supports a direct role for vasopressin, through the activation of V2 receptors, in the development and progression of CKD, including DKD [6–16]. Impaired kidney function may aggravate other cardiovascular risk factors such as hypertension, oxidative stress, insulin resistance, dyslipidemia, body fat distribution, inflammation, and arterial calcification [44–46]. Thus, the association of copeptin with CVD could be accounted for, at least in part, by the deleterious effects of vasopressin on the kidney. However, vasopressin has many other physiological actions in multiple systems in addition to its well-defined V2R-mediated role in the control of fluid homeostasis and urine concentration [47]. Vasopressin induces platelet aggregation and has a vasoconstrictor effect on vascular smooth muscle cells via V1aR [48]. Vasopressin stimulates gluconeogenesis and glycogenolysis through the activation of hepatic V1aR [48–51], and the release either of glucagon or insulin, depending on concomitant extracellular glucose levels, through the activation of V1bR in pancreatic islets [52]. It also stimulates the release of adrenocorticotropic hormone and cortisol through activation of pituitary V1bR [53, 54]. There is now strong experimental evidence that vasopressin plays a role in glucose homeostasis, and that high vasopressin levels are a risk factor for the metabolic syndrome and diabetes [55]. Acute infusion of vasopressin in rodents [56, 57] and in healthy people [58] induces a transient rise in hepatic glucose production and in blood glucose levels. Chronic intraperitoneal infusion of vasopressin in rodents induces an increase in gluconeogenesis and fasting plasma glucose, and promotes hyperinsulinemia and glucose intolerance [51, 57]. These effects are blunted by the co-administration of a V1aR antagonist. Moreover, low circulating levels of vasopressin obtained by increasing daily water intake were associated with a drastic reduction in liver steatosis in obese Zucker rats [51]. Plasma copeptin was shown to be positively associated with insulin resistance, the metabolic syndrome and with the incidence or the prevalence of type 2 diabetes in the general population [32, 33, 35, 59–62]. Taken together, the available data suggest that vasopressin has several potential dysmetabolic and pro-atherogenic effects that could explain the association of copeptin with CVD.

There are limitations of our study to acknowledge. We have measured copeptin as a surrogate of vasopressin. However, plasma concentrations of vasopressin and copeptin correlate over a wide range of plasma and/or urine osmolalities [34], and the correlation seems relatively stable for eGFR > 28 ml/min/1.73 m^2^ [63]. Only 78 SURDIAGENE participants had baseline eGFR below this threshold, and their exclusion had only a minimal impact on the results (data not shown). Half of the patients received ramipril during follow-up in the original DIABHYCAR study [22], and this drug may influence blood pressure. However, we observed no interaction between copeptin and study treatment (ramipril vs placebo) in any of our DIABHYCAR results, and adjustment for ramipril treatment had no impact on the results. Finally, because of the observational design, our study does not allow any direct demonstration of a causal relationship between vasopressin and CVD.

## Conclusions

The present investigation confirmed in two independent cohorts the association between plasma copeptin and cardiovascular risk in people with type 2 diabetes. Moreover, it extends this observation by showing that the cardiovascular risk associated with high plasma copeptin cannot be accounted for solely by the association of copeptin with markers of kidney disease. Plasma copeptin could possibly help to target patients with high risk of DKD and CVD development and progression. Increased plasma osmolality is the main stimulus for vasopressin and copeptin secretion, which are thus strongly dependent on the hydration status. It remains to be established if an effective reduction of vasopressin secretion or action, achieved by increased water intake or by treatment with vasopressin receptor antagonists (vaptans), could improve the cardiometabolic and kidney risks in people with type 2 diabetes.

## Additional file

**Additional file 1: Figure S1.** Plasma copeptin by KDIGO eGFR categories and by UAC categories at baseline. **Table S1.** Clinical characteristics at baseline by tertiles of plasma copeptin. **Table S2.** Risk of individual cardiovascular outcomes during follow-up by tertiles of plasma copeptin at baseline—DIABHYCAR and SURDIAGENE pooled data. **Table S3.** Rapid kidney function decline during follow-up by tertiles of plasma copeptin at baseline. **Table S4.** SURGENE cohort—Kidney outcome during the follow-up by tertiles of plasma copeptin at baseline. **Table S5.** Cardiovascular events during follow-up by tertiles of plasma copeptin at baseline—DIABHYCAR and SURDIAGENE pooled data. **Additional information**. Centers and staff involved in SURDIAGENE recruitment and adjudication.

## Abbreviations

ACE: angiotensin converting enzyme
ANCOVA: analysis of covariance
ANOVA: analysis of variance
BMI: body mass index
CHF: congestive heart failure
CI: confidence interval
CKD: chronic kidney disease
CV: coefficient of variation
CVD: cardiovascular disease
DKD: diabetic kidney disease
eGFR: estimated glomerular filtration rate
ESRD: end-stage renal disease
HR: hazard ratio
KDIGO: kidney disease improving global outcomes
OR: odds ratio
UAC: urinary albumin concentration
