# Supplementary material for: Plasma copeptin, kidney disease, and risk for cardiovascular morbidity and mortality in two cohorts of type 2 diabetes
Source: Cardiovasc Diabetol. 2018 Aug 2;17:110. doi: 10.1186/s12933-018-0753-5 (PMC6071392; doi:10.1186/s12933-018-0753-5)
Supplement: Supplementary file 1 — Additional file 1: Figure S1. Plasma copeptin by KDIGO eGFR categories and by UAC categories at baseline. Table S1. Clinical characteristics at baseline by tertiles of plasma copeptin. Table S2. Risk of individual cardiovascular outcomes during follow-up by tertiles of plasma copeptin at baseline—DIABHYCAR and SURDIAGENE pooled data. Table S3. Rapid kidney function decline during follow-up by tertiles of plasma copeptin at baseline. Table S4. SURGENE cohort—Kidney outcome during the follow-up by tertiles of plasma copeptin at baseline. Table S5. Cardiovascular events during follow-up by tertiles of plasma copeptin at baseline—DIABHYCAR and SURDIAGENE pooled data. Additional information. Centers and staff involved in SURDIAGENE recruitment and adjudication. [file 12933_2018_753_MOESM1_ESM.pdf]

G. Velho and coworkers

Plasma copeptin, kidney disease, and risk for cardiovascular morbidity and mortality in two cohorts of type 2 diabetes

Cardiovascular Diabetology, 2018, doi.org/10.1186/s12933-018-0753-5

Additional File 1: Supplementary Material

Figure S1. Plasma copeptin by KDIGO eGFR categories and by UAC categories at baseline.

Table S1. Clinical characteristics at baseline by tertiles of plasma copeptin.

Table S2. Risk of individual cardiovascular outcomes during follow-up by tertiles of plasma copeptin at baseline - DIABHYCAR and SURDIAGENE pooled data.

Table S3. Rapid kidney function decline during follow-up by tertiles of plasma copeptin at baseline.

Table S4. SURGENE cohort – Kidney outcome during the follow-up by tertiles of plasma copeptin at baseline.

Table S5. Cardiovascular events during follow-up by tertiles of plasma copeptin at baseline - DIABHYCAR and SURDIAGENE pooled data.

Supplementary Information. Centers and staff involved in SURDIAGENE recruitment and adjudication

Figure S1: Plasma copeptin by KDIGO eGFR categories (panel A) and by UAC categories (panel B) at baseline in DIABHYCAR (black box plots) and SURDIAGENE (red box plots) cohorts. Box plots show the median and the interquartile range (IQR), and whiskers show the interval of 1.5 IQR from the lower and upper quartiles. Wilcoxon / Kruskal-Wallis test: chi-square 99.6, p<0.0001 and chi-square 26.0, p<0.0001 for the comparisons of copeptin by KDIGO eGFR categories and by UAC categories, respectively, in DIABHYCAR, and chi-square 348.6, p<0.0001 and chi-square 173.1, p<0.0001, respectively, for the comparisons in SURDIAGENE. As for the exclusion criteria of the original DIABHYCAR study, none of its participants were normoalbuminuric nor had a severely decreased eGFR (KDIGO categories G4 and G5) at baseline.

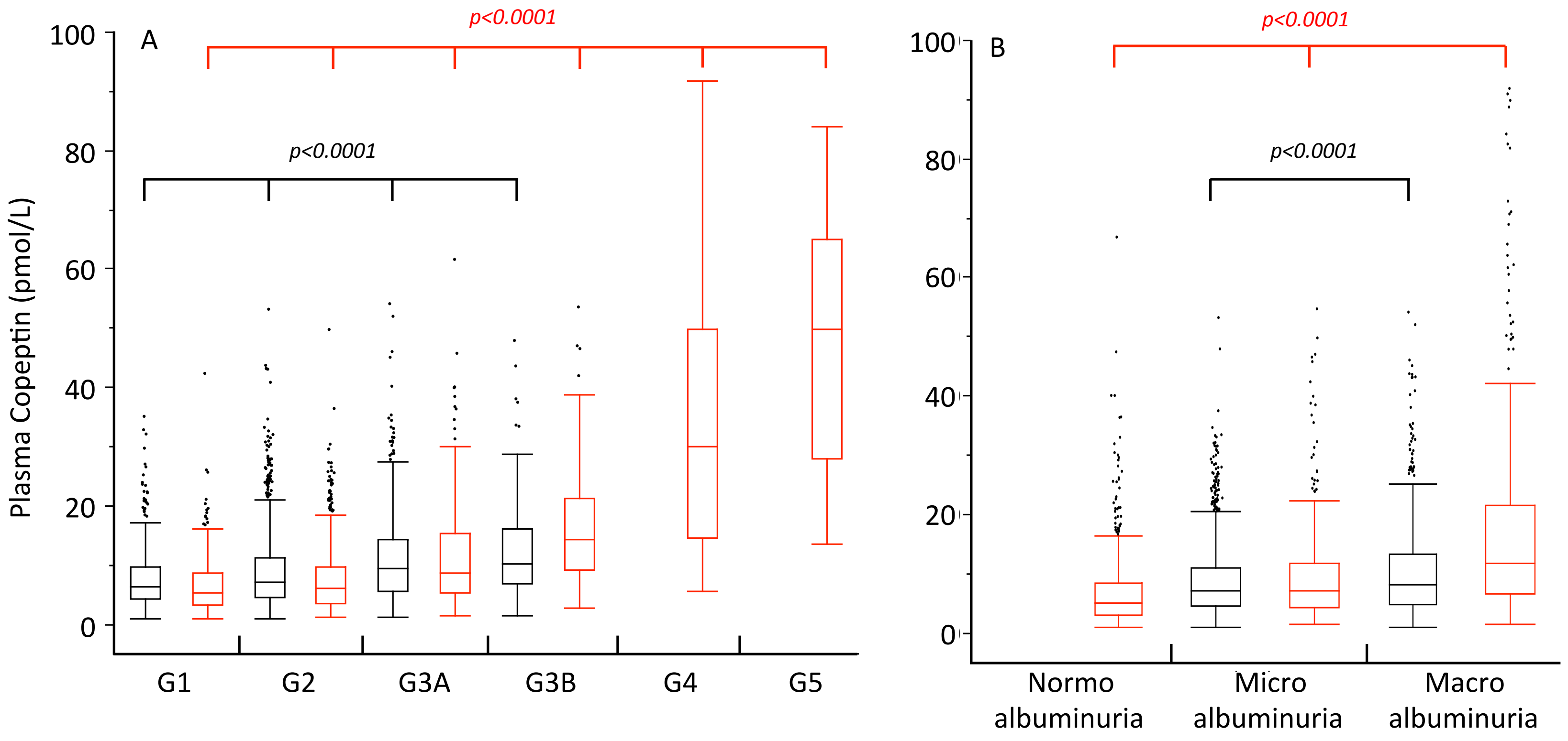

Table S1: Clinical characteristics at baseline by tertiles of plasma copeptin

	DIABHYCAR				SURDIAGENE			
	T1	T2	T3	p	T1	T2	T3	p
N	1034	1034	1030		469	469	469	
Plasma copeptin (pmol/l)*	3.7 (2.0)	7.2 (2.2)[a]	13.5 (6.5)[a,b]	<0.0001	3.1 (1.9)	6.8 (2.5) [a]	16.2 (12.2) [a,b]	<0.0001
Sex: male (%)	73	73	73	0.99	58	57	58	87
Age (y)	65 ± 8	65 ± 8	67 ± 9 [a,b]	<0.0001	63 ± 10	64 ± 10	67 ± 11 [a,b]	<0.0001
BMI (kg/m^2)	29.3 ± 4.5	29.5 ± 4.6	29.3 ± 4.8	0.45	30.9 ± 5.8	31.6 ± 6.4	31.5 ± 6.6	0.20
Duration of diabetes (y)	10 ± 8	10 ± 8	11 ± 7	0.05	14 ± 10	14 ± 10	16 ± 10 [a,b]	<0.0001
HbA1c (%)	7.7 ± 1.6	7.8 ± 1.7	8.2 ± 1.9 [a,b]	<0.0001	7.6 ± 1.3	7.9 ± 1.6 [a]	7.8 ± 1.6	0.009
HbA1c (mmol/mol)	60 ± 18	61 ± 19	65 ± 21 [a,b]	<0.0001	59 ± 15	63 ± 18	62± 18	0.01
Systolic BP (mmHg)	145 ± 14	145 ± 14	145 ± 14	0.92	131 ± 17	132 ± 17	135± 19 [a,b]	0.002
Diastolic BP (mmHg)	82 ± 8	82 ± 8	82 ± 9	0.81	71 ± 10	73 ± 11	73 ± 12	0.14
Arterial Hypertension (%)	52	55	62	<0.0001	78	84	89	<0.0001
UAC (mg/24h)*	69 (115)	74 (138) [a]	85 (196) [a,b]	<0.0001	13 (35)	23 (70) [a]	64 (419) [a,b]	<0.0001
eGFR (ml/min/1.73m^2)	78 ± 16	76 ± 17 [a]	69 ± 18 [a,b]	<0.0001	83 ± 17	77 ± 21 [a]	57 ± 28 [a,b]	<0.0001
Total cholesterol (mmol/l)	5.77 ± 1.01	5.77 ± 1.08	5.83 ± 1.11	0.42	4.70 ± 1.02	4.76 ± 1.16	4.87 ± 1.28	0.24
HDL cholesterol (mmol/l)	1.36 ± 0.36	1.32 ± 0.35	1.30 ± 0.35	0.34	1.23 ± 0.42	1.19 ± 0.40	1.16 ± 0.41 [a]	0.02
Triglycerides (mmol/l)	2.22 ± 1.58	2.13 ± 1.16	2.31 ± 1.46 [b]	0.04	1.73 ± 1.09	1.92 ± 1.34 [a]	2.12 ± 1.82 [a]	<0.0001
Previous myocardial infarction (%)	6.5	5.4	4.7	0.19	13.7	13.4	20.0	0.007
Active tobacco smoking (%)	16	13	14	0.11	12	11	8	0.15

Data expressed as mean ± SD except (*) expressed as median and interquartile range. Statistics for quantitative parameters are ANOVA with log-transformed data, except (*) Wilcoxon / Kruskal-Wallis rank sums test. Tukey Kramer HSD test following ANOVA or Wilcoxon for each pair: significantly different ($p<0.05$) from T1 (a) or T2 (b). HbA1c is expressed in % of total hemoglobin and in mmol/mol (millimoles HbA1c per mole of total hemoglobin). UAC: urinary albumin concentration. $p<0.05$ is significant.

Table S2: Risk of individual cardiovascular outcomes during follow-up by tertiles of plasma copeptin at baseline - DIABHYCAR and SURDIAGENE pooled data.

	Model 1		Model 2	
	HR (95% CI)	p	HR (95% CI)	p
Myocardial infarction				
T3 vs T1	1.96 (1.39 – 2.80)	0.0001	1.61 (1.11 – 2.36)	0.01
T2 vs T1	1.36 (0.94 – 1.98)	0.10	1.32 (0.91 – 1.93)	0.14
T3 vs T2	1.44 (1.05 – 1.9)	0.02	1.22 (0.87 – 1.71)	0.25
Coronary revascularization				
T3 vs T1	1.46 (1.15 – 1.86)	0.002	1.33 (1.03 – 1.72)	0.03
T2 vs T1	1.42 (1.12 – 1.80)	0.004	1.37 (1.08 – 1.75)	0.01
T3 vs T2	1.03 (0.83 – 1.29)	0.78	0.97 (0.77 – 1.22)	0.77
Congestive heart failure				
T3 vs T1	1.75 (1.35 – 2.28)	<0.0001	1.40 (1.06 – 1.86)	0.02
T2 vs T1	1.10 (0.83 – 1.46)	0.52	1.04 (0.78 – 1.39)	0.78
T3 vs T2	1.59 (1.24 – 2.06)	0.0002	1.34 (1.03 – 1.75)	0.03

Stroke				
T3 vs T1	1.01 (0.74 – 1.39)	0.95	0.89 (0.64 – 1.26)	0.52
T2 vs T1	0.94 (0.68 – 1.30)	0.72	0.93 (0.67 – 1.30)	0.69
T3 vs T2	1.07 (0.78 – 1.48)	0.67	0.96 (0.69 – 1.34)	0.80
Cardiovascular death				
T3 vs T1	1.83 (1.46 – 2.31)	<0.0001	1.31 (1.02 – 1.68)	0.04
T2 vs T1	1.52 (1.20 – 1.92)	0.0005	1.45 (1.14 – 1.85)	0.002
T3 vs T2	1.21 (0.99 – 1.49)	0.07	0.90 (0.72 – 1.23)	0.34

Hazards ratio (HR) computed by Cox proportional hazards survival regression analysis. Model 1: adjusted for sex, age, BMI, duration of diabetes, systolic blood pressure, arterial hypertension, HbA1c, total cholesterol, HDL-cholesterol, triglycerides, active tobacco smoking, and previous history of myocardial infarction at baseline. Model 2: model 1 plus adjustments for eGFR and UAC at baseline. $p<0.05$ is significant

Table S3: Rapid kidney function decline during follow-up by tertiles of plasma copeptin at baseline

	DIABHYCAR		SURDIAGENE	
	No rapid decline	Rapid decline	No rapid decline	Rapid decline
T1	747 (85.3%)	129 (14.7%)	345 (83.3%)	69 (16.7%)
T2	725 (84.6%)	132 (15.4%)	308 (80.4%)	75 (19.6%)
T3	636 (78.8%)	171 (21.2%)	270 (70.3%)	114 (29.7%)
	OR (95% CI)	p	OR (95% CI)	p
T3 vs T1	1.61 (1.24 – 2.11)	0.0004	2.32 (1.58 – 3.43)	<0.0001
T2 vs T1	1.06 (0.81 – 1.39)	0.67	1.39 (0.90 – 1.90)	0.16
T3 vs T2	1.52 (1.17 – 1.99)	0.002	1.77 (1.22 – 2.58)	0.002
Log_e[copeptin]	1.46 (1.23 – 1.74)	<0.0001	1.69 (1.36 – 2.10)	<0.0001

Rapid kidney function decline during follow-up defined by a slope of eGFR steeper than -5 mL/min/1.73 m^2 per year. Data expressed as number of cases and (%) by line. Odds ratio (OR) computed by logistic regression analysis for tertiles of plasma copeptin, and for 1 unit of log_e[copeptin]. Analyses adjusted for sex, age, eGFR and UAC at baseline, and duration of follow-up. $p<0.05$ is significant.

Table S4: SURGENE cohort – Kidney outcome during follow-up by tertiles of plasma copeptin at baseline

	No outcomes	Kidney outcomes	
T1	455 (97.0%)	14 (3.0%)	
T2	450 (95.9%)	19 (4.1%)	
T3	390 (83.2%)	79 (16.8%)	
		HR (95% CI)	p
T3 vs T1		3.19 (1.74 – 6.18)	0.0001
T2 vs T1		1.35 (0.68 – 2.75)	0.68
T3 vs T2		2.36 (1.38 – 4.18)	0.001
Log_e[copeptin]		1.42 (1.04 – 1.94)	0.03

Kidney outcome defined as doubling of serum creatinine or the development of ESRD (requirement of hemodialysis or kidney transplantation) during follow-up. Data expressed as number of cases and (%) by line. Hazards ratio (HR) computed by Cox proportional hazards survival regression analysis for tertiles of plasma copeptin, and for 1 unit of log_e[copeptin]. Analyses adjusted for sex, age, duration of diabetes, eGFR and UAC at baseline. $p<0.05$ is significant.

Table S5: Cardiovascular events during follow-up by tertiles of plasma copeptin at baseline - DIABHYCAR and SURDIAGENE pooled data.

	No events	Cardiovascular events	
T1	1212 (80.6%)	291 (19.4%)	
T2	1150 (76.53%)	353 (23.5%)	
T3	1054 (70.3%)	446 (29.7%)	
Model 1	HR (95% CI)		p
T3 vs T1	1.66 (1.42 – 1.94)		<0.0001
T2 vs T1	1.35 (1.16 – 1.59)		0.0002
T3 vs T2	1.23 (1.06 – 1.41)		0.005
Log_e[copeptin]	1.38 (1.27 – 1.51)		<0.0001
Model 2	HR (95% CI)		p
T3 vs T1	1.34 (1.14 – 1.58)		0.0004
T2 vs T1	1.29 (1.10 – 1.51)		0.001
T3 vs T2	1.04 (0.90 – 1.21)		0.60
Log_e[copeptin]	1.17 (1.07 – 1.29)		0.0009

Data expressed as number of cases and (%) by line. Hazards ratio (HR) computed by Cox proportional hazards survival regression analysis for tertiles of plasma copeptin, and for 1 unit of log_e[copeptin]. Model 1: adjusted for cohort, sex, age, BMI, duration of diabetes, systolic blood pressure, arterial hypertension, HbA1c, total cholesterol, HDL-cholesterol, triglycerides, active tobacco smoking, and previous history of myocardial infarction at baseline. Model 2: model 1 plus adjustments for KDIGO eGFR category and UAC category (normo, micro or macroalbuminuria) at baseline. p(interaction)=0.21 for copeptin tertiles / KDIGO eGFR category; p(interaction)=0.63 for copeptin tertiles / UAC category. $p<0.05$ is significant.

Supplementary Information

Centers and staff involved in SURDIAGENE recruitment and adjudication

Patients and clinicians
All patients included and followed in the cohort study are warmly thanked for their kind participation to this research. Their GPs are acknowledged for their help to collect clinical information.

Center organisation
Recruiting physicians: Samy Hadjadj (Coordinator), Frédérique Duengler, Louis Labbé, Aurélie Miot, Xavier Piguel, Stéphanie Laugier-Robiole, Florence Torremocha, Pierre-Jean Saulnier, Richard Maréchaud.

Secretarial and technical assistance: Cécile Demer and all the staff from the department of endocrinology, diabetology (recruitment) and Sonia Brishoual and the staff of the INSERM CIC 802 (biobanking and data management). Gérard Mauco (Department of biochemistry, CHU Poitiers) and Thierry Hauet (INSERM U1082, CHU Poitiers) are acknowledged for helping in biological determinations.

Baseline data case review
All patient records were reviewed to ascertain the following points: type 2 diabetes, diabetic kidney disease, diabetic retinopathy and cardiovascular disease. The clinicians involved in this process are warmly thanked here: Daniel Herpin & Philippe Sosner (Cardiology), Frank Bridoux (Nephrology), Helene Manic (Ophthalmology) and Samy Hadjadj (Diabetology).

Adjudication procedure
Case inquiry – Samy Hadjadj (Coordinator), Sonia Brishoual, Céline Divoy, Cécile Demer, Aurélie Miot, Xavier Piguel, Florence Torremocha, Nathalie Fauvergue, Séverin Carasson, Pierre-Jean Saulnier, Philippe Sosner

Local coordination: Stéphanie Ragot (coordinator & biostatistician), Fabrice Lebel (Data manager), Elise Gand (Data management and biostatistics)

Adjudication committee: Jean-Michel Halimi (Chairman, Tours), Gregory Ducrocq (Paris Bichat), Charlotte Hulin (Poitiers), Pierre Llatty (Poitiers), David Montaigne (Lille), Vincent Rigalleau (Bordeaux), Ronan Roussel (Paris Bichat), Philippe Zaoui (Grenoble).

Quality control (INSERM CIC 0802): Pierre-Jean Saulnier, Astrid de Hautecloque, Frederike Limousi, Nathalie Fauvergue, Sofia Hermann, Sonia Brishoual
